# Ethyl 7-oxo-7*H*-benzo[*de*]imidazo[5,1-*a*]iso­quinoline-11-carboxyl­ate–tri­fluoro­acetic acid (1/1)

**DOI:** 10.1107/S1600536813013834

**Published:** 2013-05-31

**Authors:** Robert T. Stibrany, Joseph A. Potenza

**Affiliations:** a Department of Chemistry and Chemical Biology, Rutgers, The State University of New Jersey, 610 Taylor Road, Piscataway, New Jersey 08854, USA

## Abstract

The structure of the title tri­fluoro­acetic acid adduct, C_17_H_12_N_2_O_3_·C_2_HF_3_O_2_, contains a tri­fluoro­acetic acid mol­ecule hydrogen bonded to the imine N atom of the imidazole ring of a nearly planar four-fused-ring system (r.m.s. deviatiation = 0.013 Å). The carb­oxy­lic acid group of the triflouro­acetic acid mol­ecule is twisted with respect to the mean plane of the four-fused-ring sytem by 75.9 (2)°. A short intra­molecular C—H⋯O hydrogen bond occurs. In the crystal, the adduct mol­ecules are arranged into stacks along the *b* axis *via* π–π inter­actions between imidazole rings and between imidazole and one of the benzene rings [centroid–centroid distances 3.352 (2) and 3.485 (2) Å, respectively]. Molecules are linked *via* C—H⋯O hydrogen bonds, forming an alternating polymeric head-to-head/tail-to-tail stepped chain approximately along the *a*-axis direction and tilted on an axis bisecting the *b* and *c* axes.

## Related literature

For ^19^F NMR studies of related compounds, see: Stibrany (2003 ▶). For polymerization studies, see: Stibrany *et al.* (2003 ▶). For their use as agents to study electron transfer, see: Knapp *et al.* (1990 ▶). For related structures, see: Baugh *et al.* (2006 ▶); Stibrany (2003 ▶); Stibrany *et al.* (2002 ▶, 2004 ▶); Stibrany & Potenza (2008 ▶, 2009 ▶); Gorun *et al.* (1996 ▶).
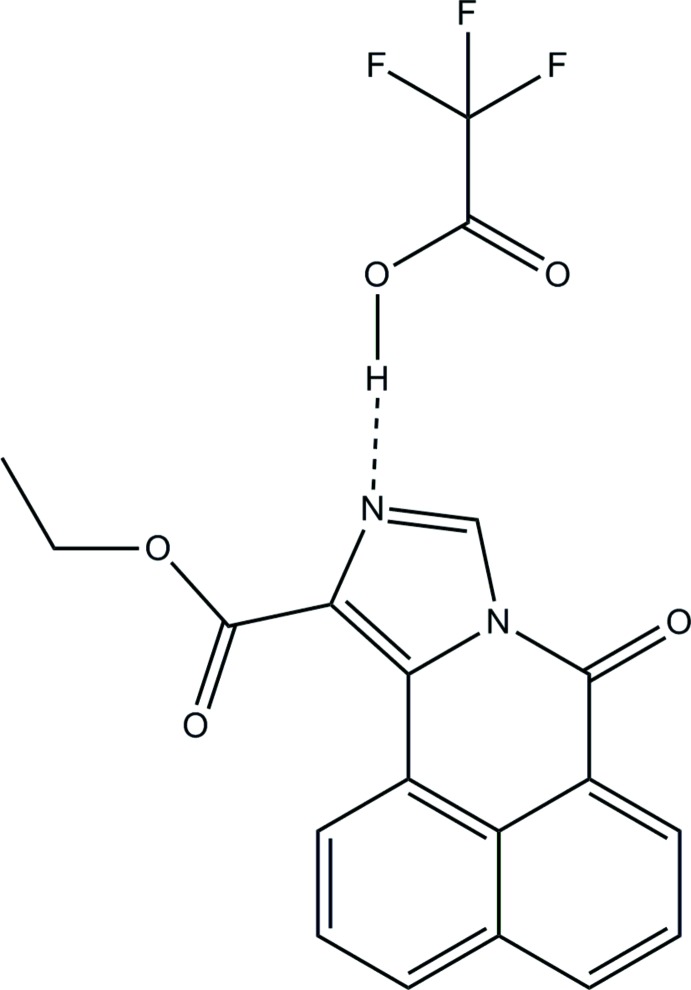


## Experimental

### 

#### Crystal data

C_17_H_12_N_2_O_3_·C_2_HF_3_O_2_
*M* *_r_* = 406.31
Triclinic, 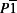

*a* = 7.642 (3) Å
*b* = 8.111 (4) Å
*c* = 14.043 (6) Å
α = 97.539 (8)°
β = 98.055 (8)°
γ = 92.695 (8)°
*V* = 852.6 (6) Å^3^
*Z* = 2
Mo *K*α radiation
μ = 0.14 mm^−1^
*T* = 100 K
0.48 × 0.10 × 0.07 mm

#### Data collection

Bruker SMART CCD area-detector diffractometer
Absorption correction: multi-scan (*SADABS*; Bruker, 2000 ▶; Blessing, 1995 ▶) *T*_min_ = 0.711, *T*_max_ = 1.00
7689 measured reflections
3380 independent reflections
2642 reflections with *I* > 2σ(*I*)
*R*_int_ = 0.032

#### Refinement

*R*[*F* ^2^ > 2σ(*F* ^2^)] = 0.050
*wR*(*F* ^2^) = 0.140
*S* = 1.00
3380 reflections
267 parameters
H atoms treated by a mixture of independent and constrained refinement
Δρ_max_ = 0.42 e Å^−3^
Δρ_min_ = −0.28 e Å^−3^

### 

Data collection: *SMART* (Bruker, 2000 ▶); cell refinement: *SAINT* (Bruker, 2000 ▶); data reduction: *SAINT*; program(s) used to solve structure: *SHELXS97* (Sheldrick, 2008 ▶); program(s) used to refine structure: *SHELXL97* (Sheldrick, 2008 ▶); molecular graphics: *ORTEPIII* (Burnett & Johnson, 1996 ▶) and *ORTEP-3 for Windows* (Farrugia, 2012 ▶); software used to prepare material for publication: *SHELXTL* (Sheldrick, 2008 ▶).

## Supplementary Material

Crystal structure: contains datablock(s) I, global. DOI: 10.1107/S1600536813013834/gk2567sup1.cif

Structure factors: contains datablock(s) I. DOI: 10.1107/S1600536813013834/gk2567Isup2.hkl

Additional supplementary materials:  crystallographic information; 3D view; checkCIF report

## Figures and Tables

**Table 1:** Hydrogen-bond geometry (Å, °)

*D*—H⋯*A*	*D*—H	H⋯*A*	*D*⋯*A*	*D*—H⋯*A*
C22—H22⋯O11	0.93	2.15	2.981 (3)	148
O2—H2*O*⋯N13	1.03 (3)	1.58 (3)	2.597 (2)	170 (3)
C13—H13⋯O30^i^	0.93	2.28	3.143 (3)	154
C23—H23⋯O11^ii^	0.93	2.46	3.320 (3)	155

Symmetry codes: (i) 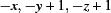; (ii) 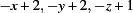.

**Fig. 1.**
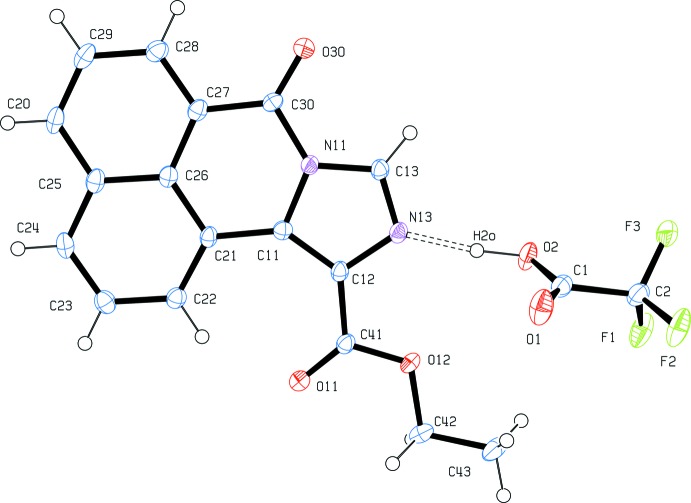
The molecular structure of the title compound showing the atom-numbering scheme. Displacement ellipsoids are shown at the 40% probability level. H atoms are shown as spheres of arbitrary radius.

**Fig. 2.**
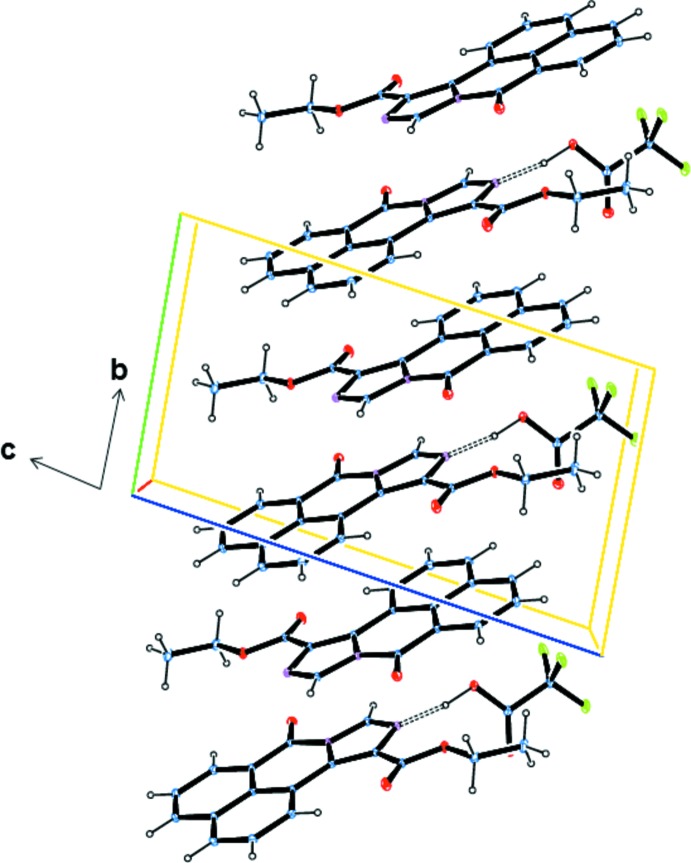
Packing of the π-π stacked imidazole dimers approximately along the b axis in the unit cell viewed approximately down the a axis.

## supplementary crystallographic information

### Comment

The title compound, Fig. 1, was isolated as part of our long-term interest in the chemistry of bis (imidazoles), bis(benzimidazoles), and their complexes with metal ions. These species have demonstrated their usefulness as proton sponges (Stibrany *et al.*, 2002), geometrically constraining ligands (Stibrany *et al.*, 2004), agents to study electron transfer (Knapp *et al.*, 1990), polymerization catalysts (Stibrany *et al.*, 2003; Baugh *et al.*, 2006), ^19^F NMR polymerization catalyst probes (Stibrany, 2003), and in the formation of metal-organic copolymers (Stibrany & Potenza, 2008). Previously, we have shown that 1-methylbenzimidazole can be used in the synthesis of bis(benzimidazole)ketones, which were found to be useful ligands for the chelation of metals (Gorun *et al.*, 1996).

Our investigation into the synthesis of acenapthoimidazoles as building blocks for higher dentate ligands led us to attempt a similar method of preparation as for phenanthroimidazoles (Stibrany & Potenza, 2009). The title compound was isolated by chromatography as a side product of that preparation. The trifluoroacetic acid adduct [C_18_H_12_N_2_O_3_][C_2_HF_3_O_2_] contains trifluoroacetic acid molecule, hydrogen bonded to the imine nitrogen of the imidazole ring of a nearly-planar, four-fused-ring system (r.m.s. deviatiation = 0.013 Å). A carbonyl-centroid interaction is formed by C41-O11 to the centroid formed by C20, C25/C29 (-x+1, -y, -z+1) with a O11-Cg distance of 3.567 (2) Å and a C41-O11···Cg angle of 78.0 (1)°. In the space group P1 , the adduct molecules are centrosymmetrically disposed about the origin, and form π-π dimers through the imidazole rings along the *b* cell direction Fig 2. The first Cg-Cg interaction is the imidazole ring (N11, N13, C11/C13) paired to a symmetry related imidazole (-x+1, -y+1, -z+1) at a distance of 3.352 (2) Å. A second Cg-Cg interaction is formed by the imidazole ring and the centroid C21/C26 (-x+1, -y, 1-z) at a distance of 3.485 (2) Å. There are three short intermolecular hydrogen bonds and one short intramolecular hydrogen bond found in the structure and are listed in the hydrogen-bond Table 1. No additional electron density was located near N13 in the difference Fourier maps, likely due to the electron-withdrawing effect of the adjacent ethyl ester group.

### Experimental

The title compound was isolated as a minor side product in the condensation of acenaphthaquinone in place of phenanthroquinone (Stibrany & Potenza, 2009). A small yellow–orange band was isolated by chromatography on silica gel using ethyl acetate as the eluent. Two X-ray quality crystals were obtained by slow evaporation of a 10:1 *v*/*v* methanol/trifluoroacetic acid solution of the title compound.

### Refinement

Hydrogen atoms were positioned geometrically and refined using a riding model, with C—H = 0.97 (methylene), 0.96 (methyl) and 0.93 Å (aromatic), and with *U*_iso_(H) = 1.2–1.5*U*_eq_ (C). The carboxylic hydrogen atom was freely refined.

### Crystal data

**Table:**

C_17_H_12_N_2_O_3_·C_2_HF_3_O_2_	*Z* = 2
*M**_r_* = 406.31	*F*(000) = 416
Triclinic, *P*1	*D*_x_ = 1.583 Mg m^−^^3^
Hall symbol: -P 1	Mo *K*α radiation, λ = 0.71073 Å
*a* = 7.642 (3) Å	Cell parameters from 800 reflections
*b* = 8.111 (4) Å	θ = 2.5–26.0°
*c* = 14.043 (6) Å	µ = 0.14 mm^−^^1^
α = 97.539 (8)°	*T* = 100 K
β = 98.055 (8)°	Spike, yellow
γ = 92.695 (8)°	0.48 × 0.10 × 0.07 mm
*V* = 852.6 (6) Å^3^	

### Data collection

**Table:**

Bruker SMART CCD area-detector diffractometer	3380 independent reflections
Radiation source: fine-focus sealed tube	2642 reflections with *I* > 2σ(*I*)
Graphite monochromator	*R*_int_ = 0.032
φ and ω scans	θ_max_ = 26.2°, θ_min_ = 2.5°
Absorption correction: multi-scan (*SADABS*; Bruker, 2000; Blessing, 1995)	*h* = −9→9
*T*_min_ = 0.711, *T*_max_ = 1.00	*k* = −10→10
7689 measured reflections	*l* = −17→17

### Refinement

**Table:**

Refinement on *F*^2^	Primary atom site location: structure-invariant direct methods
Least-squares matrix: full	Secondary atom site location: difference Fourier map
*R*[*F*^2^ > 2σ(*F*^2^)] = 0.050	Hydrogen site location: inferred from neighbouring sites
*wR*(*F*^2^) = 0.140	H atoms treated by a mixture of independent and constrained refinement
*S* = 1.00	*w* = 1/[σ^2^(*F*_o_^2^) + (0.0934*P*)^2^ + 0.124*P*] where *P* = (*F*_o_^2^ + 2*F*_c_^2^)/3
3380 reflections	(Δ/σ)_max_ < 0.001
267 parameters	Δρ_max_ = 0.42 e Å^−^^3^
0 restraints	Δρ_min_ = −0.28 e Å^−^^3^

### Special details

**Table:**

Geometry. All e.s.d.'s (except the e.s.d. in the dihedral angle between two l.s. planes) are estimated using the full covariance matrix. The cell e.s.d.'s are taken into account individually in the estimation of e.s.d.'s in distances, angles and torsion angles; correlations between e.s.d.'s in cell parameters are only used when they are defined by crystal symmetry. An approximate (isotropic) treatment of cell e.s.d.'s is used for estimating e.s.d.'s involving l.s. planes.
Refinement. Refinement of *F*^2^ against ALL reflections. The weighted *R*-factor *wR* and goodness of fit *S* are based on *F*^2^, conventional *R*-factors *R* are based on *F*, with *F* set to zero for negative *F*^2^. The threshold expression of *F*^2^ > σ(*F*^2^) is used only for calculating *R*-factors(gt) *etc*. and is not relevant to the choice of reflections for refinement. *R*-factors based on *F*^2^ are statistically about twice as large as those based on *F*, and *R*- factors based on ALL data will be even larger.

### Fractional atomic coordinates and isotropic or equivalent isotropic displacement parameters (Å^2^)

**Table:**

	*x*	*y*	*z*	*U*_iso_*/*U*_eq_	
F1	0.25760 (19)	0.12397 (17)	0.05463 (9)	0.0435 (4)	
F2	0.0782 (2)	0.27375 (17)	−0.01973 (9)	0.0460 (4)	
F3	−0.0070 (2)	0.13711 (19)	0.08785 (10)	0.0550 (5)	
O1	0.1872 (2)	0.51438 (18)	0.12697 (11)	0.0390 (4)	
O2	0.2409 (2)	0.32127 (17)	0.22686 (10)	0.0300 (4)	
O11	0.79755 (19)	0.70261 (18)	0.38002 (10)	0.0310 (4)	
O12	0.60779 (18)	0.52898 (16)	0.27167 (9)	0.0242 (3)	
O30	0.08434 (17)	0.69206 (16)	0.60128 (9)	0.0250 (3)	
N11	0.3148 (2)	0.69116 (18)	0.51474 (11)	0.0185 (3)	
N13	0.3464 (2)	0.54546 (18)	0.37627 (11)	0.0201 (4)	
C1	0.1900 (3)	0.3743 (2)	0.14513 (13)	0.0242 (4)	
C2	0.1289 (3)	0.2258 (3)	0.06570 (14)	0.0276 (5)	
C11	0.4856 (2)	0.7380 (2)	0.49858 (13)	0.0183 (4)	
C12	0.5023 (2)	0.6433 (2)	0.41081 (12)	0.0189 (4)	
C13	0.2372 (2)	0.5763 (2)	0.43870 (12)	0.0199 (4)	
H13	0.1230	0.5272	0.4325	0.024*	
C20	0.5334 (3)	1.1120 (2)	0.80732 (13)	0.0260 (5)	
H20	0.5971	1.1923	0.8543	0.031*	
C21	0.5911 (2)	0.8628 (2)	0.57107 (12)	0.0185 (4)	
C22	0.7608 (2)	0.9217 (2)	0.56293 (13)	0.0217 (4)	
H22	0.8127	0.8794	0.5096	0.026*	
C23	0.8557 (3)	1.0438 (2)	0.63361 (14)	0.0248 (4)	
H23	0.9689	1.0822	0.6262	0.030*	
C24	0.7837 (3)	1.1071 (2)	0.71340 (14)	0.0247 (4)	
H24	0.8483	1.1877	0.7599	0.030*	
C25	0.6110 (3)	1.0504 (2)	0.72550 (13)	0.0224 (4)	
C26	0.5131 (2)	0.9275 (2)	0.65349 (13)	0.0193 (4)	
C27	0.3396 (3)	0.8738 (2)	0.66789 (13)	0.0205 (4)	
C28	0.2685 (3)	0.9367 (2)	0.74925 (13)	0.0241 (4)	
H28	0.1553	0.8993	0.7573	0.029*	
C29	0.3665 (3)	1.0565 (2)	0.81947 (14)	0.0281 (5)	
H29	0.3188	1.0986	0.8744	0.034*	
C30	0.2317 (2)	0.7487 (2)	0.59630 (13)	0.0191 (4)	
C41	0.6521 (2)	0.6321 (2)	0.35499 (13)	0.0206 (4)	
C42	0.7451 (3)	0.5121 (3)	0.20891 (14)	0.0274 (5)	
H42A	0.8381	0.4469	0.2358	0.033*	
H42B	0.7970	0.6210	0.2027	0.033*	
C43	0.6566 (3)	0.4257 (3)	0.11143 (14)	0.0346 (5)	
H43A	0.6112	0.3161	0.1181	0.052*	
H43B	0.7413	0.4172	0.0668	0.052*	
H43C	0.5610	0.4886	0.0872	0.052*	
H2O	0.284 (4)	0.419 (4)	0.281 (2)	0.061 (8)*	

### Atomic displacement parameters (Å^2^)

**Table:**

	*U*^11^	*U*^22^	*U*^33^	*U*^12^	*U*^13^	*U*^23^
F1	0.0536 (9)	0.0426 (8)	0.0288 (7)	0.0170 (6)	−0.0016 (6)	−0.0117 (6)
F2	0.0704 (10)	0.0429 (8)	0.0192 (6)	0.0082 (7)	−0.0094 (6)	−0.0009 (5)
F3	0.0627 (10)	0.0589 (9)	0.0352 (8)	−0.0337 (8)	0.0193 (7)	−0.0232 (7)
O1	0.0595 (11)	0.0267 (8)	0.0269 (8)	0.0030 (7)	−0.0062 (7)	0.0029 (6)
O2	0.0476 (9)	0.0231 (7)	0.0164 (7)	−0.0031 (6)	−0.0007 (6)	−0.0004 (6)
O11	0.0297 (8)	0.0378 (8)	0.0236 (7)	−0.0072 (6)	0.0089 (6)	−0.0049 (6)
O12	0.0290 (7)	0.0273 (7)	0.0157 (6)	0.0000 (6)	0.0065 (5)	−0.0020 (5)
O30	0.0243 (7)	0.0270 (7)	0.0231 (7)	−0.0040 (6)	0.0063 (5)	0.0000 (5)
N11	0.0216 (8)	0.0182 (8)	0.0153 (7)	−0.0011 (6)	0.0027 (6)	0.0016 (6)
N13	0.0258 (8)	0.0189 (8)	0.0149 (7)	−0.0004 (6)	0.0013 (6)	0.0025 (6)
C1	0.0257 (10)	0.0276 (11)	0.0189 (9)	0.0026 (8)	0.0027 (7)	0.0021 (8)
C2	0.0310 (11)	0.0327 (11)	0.0188 (10)	0.0023 (9)	0.0038 (8)	0.0023 (8)
C11	0.0205 (9)	0.0189 (9)	0.0162 (9)	0.0016 (7)	0.0023 (7)	0.0049 (7)
C12	0.0228 (9)	0.0183 (9)	0.0149 (8)	0.0000 (7)	0.0002 (7)	0.0027 (7)
C13	0.0229 (9)	0.0201 (9)	0.0156 (9)	−0.0016 (7)	0.0002 (7)	0.0029 (7)
C20	0.0382 (12)	0.0202 (10)	0.0166 (9)	0.0002 (8)	−0.0010 (8)	−0.0023 (7)
C21	0.0239 (9)	0.0167 (9)	0.0140 (8)	0.0004 (7)	−0.0002 (7)	0.0027 (7)
C22	0.0254 (10)	0.0217 (9)	0.0179 (9)	0.0017 (7)	0.0024 (7)	0.0036 (7)
C23	0.0237 (10)	0.0239 (10)	0.0259 (10)	−0.0004 (8)	−0.0004 (8)	0.0054 (8)
C24	0.0291 (11)	0.0193 (9)	0.0225 (10)	−0.0035 (8)	−0.0042 (8)	0.0009 (7)
C25	0.0306 (10)	0.0183 (9)	0.0173 (9)	0.0024 (8)	−0.0009 (8)	0.0027 (7)
C26	0.0249 (10)	0.0167 (9)	0.0160 (9)	0.0012 (7)	0.0010 (7)	0.0038 (7)
C27	0.0272 (10)	0.0191 (9)	0.0156 (9)	0.0027 (8)	0.0023 (7)	0.0041 (7)
C28	0.0291 (11)	0.0244 (10)	0.0201 (9)	0.0025 (8)	0.0064 (8)	0.0045 (7)
C29	0.0398 (12)	0.0272 (11)	0.0166 (9)	0.0052 (9)	0.0053 (8)	−0.0016 (8)
C30	0.0222 (10)	0.0209 (9)	0.0153 (8)	0.0029 (7)	0.0046 (7)	0.0039 (7)
C41	0.0277 (10)	0.0185 (9)	0.0158 (9)	0.0001 (8)	0.0036 (7)	0.0027 (7)
C42	0.0315 (11)	0.0321 (11)	0.0209 (10)	0.0041 (9)	0.0109 (8)	0.0038 (8)
C43	0.0412 (13)	0.0452 (13)	0.0183 (10)	0.0076 (10)	0.0086 (9)	0.0013 (9)

### Geometric parameters (Å, º)

**Table:**

F1—C2	1.326 (3)	C20—H20	0.9300
F2—C2	1.322 (2)	C21—C22	1.386 (3)
F3—C2	1.332 (2)	C21—C26	1.426 (3)
O1—C1	1.197 (2)	C22—C23	1.402 (3)
O2—C1	1.294 (2)	C22—H22	0.9300
O2—H2O	1.03 (3)	C23—C24	1.368 (3)
O11—C41	1.210 (2)	C23—H23	0.9300
O12—C41	1.338 (2)	C24—C25	1.418 (3)
O12—C42	1.462 (2)	C24—H24	0.9300
O30—C30	1.211 (2)	C25—C26	1.425 (3)
N11—C13	1.368 (2)	C26—C27	1.425 (3)
N11—C11	1.399 (2)	C27—C28	1.381 (3)
N11—C30	1.423 (2)	C27—C30	1.463 (3)
N13—C13	1.302 (3)	C28—C29	1.397 (3)
N13—C12	1.390 (2)	C28—H28	0.9300
C1—C2	1.537 (3)	C29—H29	0.9300
C11—C12	1.390 (3)	C42—C43	1.506 (3)
C11—C21	1.461 (2)	C42—H42A	0.9700
C12—C41	1.475 (3)	C42—H42B	0.9700
C13—H13	0.9300	C43—H43A	0.9600
C20—C29	1.373 (3)	C43—H43B	0.9600
C20—C25	1.410 (3)	C43—H43C	0.9600
			
C1—O2—H2O	111.3 (16)	C23—C24—C25	120.26 (17)
C41—O12—C42	115.50 (15)	C23—C24—H24	119.9
C13—N11—C11	108.78 (15)	C25—C24—H24	119.9
C13—N11—C30	124.09 (15)	C20—C25—C24	121.70 (17)
C11—N11—C30	127.13 (15)	C20—C25—C26	118.99 (18)
C13—N13—C12	107.70 (15)	C24—C25—C26	119.30 (18)
O1—C1—O2	129.19 (18)	C25—C26—C27	117.75 (17)
O1—C1—C2	120.89 (18)	C25—C26—C21	119.44 (17)
O2—C1—C2	109.92 (17)	C27—C26—C21	122.81 (16)
F2—C2—F1	107.05 (17)	C28—C27—C26	121.52 (17)
F2—C2—F3	107.57 (17)	C28—C27—C30	118.02 (18)
F1—C2—F3	107.45 (18)	C26—C27—C30	120.46 (17)
F2—C2—C1	112.15 (17)	C27—C28—C29	120.05 (19)
F1—C2—C1	111.43 (16)	C27—C28—H28	120.0
F3—C2—C1	110.95 (16)	C29—C28—H28	120.0
C12—C11—N11	103.93 (15)	C20—C29—C28	119.86 (19)
C12—C11—C21	138.42 (17)	C20—C29—H29	120.1
N11—C11—C21	117.65 (16)	C28—C29—H29	120.1
C11—C12—N13	109.43 (16)	O30—C30—N11	119.32 (16)
C11—C12—C41	130.92 (17)	O30—C30—C27	126.38 (17)
N13—C12—C41	119.64 (16)	N11—C30—C27	114.30 (16)
N13—C13—N11	110.16 (16)	O11—C41—O12	123.27 (17)
N13—C13—H13	124.9	O11—C41—C12	125.94 (17)
N11—C13—H13	124.9	O12—C41—C12	110.78 (16)
C29—C20—C25	121.82 (17)	O12—C42—C43	106.77 (16)
C29—C20—H20	119.1	O12—C42—H42A	110.4
C25—C20—H20	119.1	C43—C42—H42A	110.4
C22—C21—C26	119.02 (16)	O12—C42—H42B	110.4
C22—C21—C11	123.35 (17)	C43—C42—H42B	110.4
C26—C21—C11	117.63 (17)	H42A—C42—H42B	108.6
C21—C22—C23	121.24 (18)	C42—C43—H43A	109.5
C21—C22—H22	119.4	C42—C43—H43B	109.5
C23—C22—H22	119.4	H43A—C43—H43B	109.5
C24—C23—C22	120.73 (19)	C42—C43—H43C	109.5
C24—C23—H23	119.6	H43A—C43—H43C	109.5
C22—C23—H23	119.6	H43B—C43—H43C	109.5
			
O1—C1—C2—F2	0.6 (3)	C20—C25—C26—C27	0.5 (3)
O2—C1—C2—F2	−178.97 (17)	C24—C25—C26—C27	−179.78 (16)
O1—C1—C2—F1	120.6 (2)	C20—C25—C26—C21	−179.23 (16)
O2—C1—C2—F1	−59.0 (2)	C24—C25—C26—C21	0.5 (3)
O1—C1—C2—F3	−119.7 (2)	C22—C21—C26—C25	−0.1 (3)
O2—C1—C2—F3	60.7 (2)	C11—C21—C26—C25	−179.51 (16)
C13—N11—C11—C12	−0.7 (2)	C22—C21—C26—C27	−179.83 (16)
C30—N11—C11—C12	178.48 (16)	C11—C21—C26—C27	0.8 (3)
C13—N11—C11—C21	179.02 (15)	C25—C26—C27—C28	−0.7 (3)
C30—N11—C11—C21	−1.8 (3)	C21—C26—C27—C28	178.95 (17)
N11—C11—C12—N13	0.52 (19)	C25—C26—C27—C30	179.58 (16)
C21—C11—C12—N13	−179.2 (2)	C21—C26—C27—C30	−0.7 (3)
N11—C11—C12—C41	−178.54 (17)	C26—C27—C28—C29	0.4 (3)
C21—C11—C12—C41	1.8 (4)	C30—C27—C28—C29	−179.91 (17)
C13—N13—C12—C11	−0.1 (2)	C25—C20—C29—C28	−0.5 (3)
C13—N13—C12—C41	179.07 (16)	C27—C28—C29—C20	0.2 (3)
C12—N13—C13—N11	−0.4 (2)	C13—N11—C30—O30	1.5 (3)
C11—N11—C13—N13	0.7 (2)	C11—N11—C30—O30	−177.63 (16)
C30—N11—C13—N13	−178.53 (15)	C13—N11—C30—C27	−179.09 (15)
C12—C11—C21—C22	0.7 (3)	C11—N11—C30—C27	1.8 (3)
N11—C11—C21—C22	−178.98 (16)	C28—C27—C30—O30	−0.8 (3)
C12—C11—C21—C26	180.0 (2)	C26—C27—C30—O30	178.88 (17)
N11—C11—C21—C26	0.4 (2)	C28—C27—C30—N11	179.81 (15)
C26—C21—C22—C23	−0.5 (3)	C26—C27—C30—N11	−0.5 (2)
C11—C21—C22—C23	178.86 (16)	C42—O12—C41—O11	−2.9 (3)
C21—C22—C23—C24	0.7 (3)	C42—O12—C41—C12	178.27 (15)
C22—C23—C24—C25	−0.3 (3)	C11—C12—C41—O11	3.7 (3)
C29—C20—C25—C24	−179.62 (18)	N13—C12—C41—O11	−175.24 (18)
C29—C20—C25—C26	0.1 (3)	C11—C12—C41—O12	−177.52 (18)
C23—C24—C25—C20	179.44 (17)	N13—C12—C41—O12	3.5 (2)
C23—C24—C25—C26	−0.3 (3)	C41—O12—C42—C43	−166.99 (16)

### Hydrogen-bond geometry (Å, º)

**Table:**

*D*—H···*A*	*D*—H	H···*A*	*D*···*A*	*D*—H···*A*
C22—H22···O11	0.93	2.15	2.981 (3)	148
O2—H2*O*···N13	1.03 (3)	1.58 (3)	2.597 (2)	170 (3)
C13—H13···O30^i^	0.93	2.28	3.143 (3)	154
C23—H23···O11^ii^	0.93	2.46	3.320 (3)	155

Symmetry codes: (i) −*x*, −*y*+1, −*z*+1; (ii) −*x*+2, −*y*+2, −*z*+1.
